# Decoding Analyses Show Dynamic Waxing and Waning of Event-Related Potentials in Coma Patients

**DOI:** 10.3390/brainsci15020189

**Published:** 2025-02-13

**Authors:** Adianes Herrera-Diaz, Rober Boshra, Richard Kolesar, Netri Pajankar, Paniz Tavakoli, Chia-Yu Lin, Alison Fox-Robichaud, John F. Connolly

**Affiliations:** 1 Department of Psychology, Georgia State University, Atlanta, GA 30303, USA; aherreradiaz@gsu.edu; 2 Georgia State/Georgia Tech Center for Advanced Brain Imaging, Atlanta, GA 30318, USA; 3 Princeton Neuroscience Institute, Princeton University, Princeton, NJ 08544, USA; rboshra@princeton.edu; 4 Department of Anesthesia, McMaster University, Hamilton, ON L8S 4L8, Canada; kolesric@hhsc.ca; 5 The Athinoula A. Martinos Center for Biomedical Imaging, Charlestown, MA 02129, USA; netrijayant@gmail.com; 6 Advanced Research in Experimental and Applied Linguistics, McMaster University, Hamilton, ON L8S 4L8, Canada; ptavakol05@gmail.com; 7 Centre for Surveillance, Integrated Insights and Risk Assessment, Data, Surveillance and Foresight Branch, Public Health Agency of Canada, Ottawa, ON K1A 0K9, Canada; chiayu.a.lin@gmail.com; 8 Department of Medicine, McMaster University, Hamilton, ON L8S 4L8, Canada; afoxrob@mcmaster.ca; 9 Critical Care Medicine, Hamilton Health Sciences, Hamilton, ON L8L 0A4, Canada; 10 School of Biomedical Engineering, McMaster University, Hamiton, ON L8S 4L8, Canada; 11 Department of Psychology, Neuroscience & Behaviour, McMaster University, Hamiton, ON L8S 4L8, Canada; 12 VoxNeuro, Inc., Toronto, ON M5H 3T9, Canada; 13 VoxNeuro USA, Inc., Cambridge, MA 02142, USA

**Keywords:** event-related potentials, ERP, coma, disorders of consciousness, multivariate decoding, MVPA, mismatch negativity (MMN)

## Abstract

**Background/Objectives**: Coma prognosis is challenging, as patient presentation can be misleading or uninformative when using behavioral assessments only. Event-related potentials have been shown to provide valuable information about a patient’s chance of survival and emergence from coma. Our prior work revealed that the mismatch negativity (MMN) in particular waxes and wanes across 24 h in some coma patients. This “cycling” aspect of the presence/absence of neurophysiological responses may require fine-grained tools to increase the chances of detecting levels of neural processing in coma. This study implements multivariate pattern analysis (MVPA) to automatically quantify patterns of neural discrimination between duration deviant and standard tones over time at the single-subject level in seventeen healthy controls and in three comatose patients. **Methods**: One EEG recording, containing up to five blocks of an auditory oddball paradigm, was performed in controls over a 12 h period. For patients, two EEG sessions were conducted 3 days apart for up to 24 h, denoted as day 0 and day 3, respectively. MVPA was performed using a support-vector machine classifier. **Results**: Healthy controls exhibited reliable discrimination or classification performance during the latency intervals associated with MMN and P3a components. Two patients showed some intervals with significant discrimination around the second half of day 0, and all had significant results on day 3. **Conclusions**: These findings suggest that decoding analyses can accurately classify neural responses at a single-subject level in healthy controls and provide evidence of small but significant changes in auditory discrimination over time in coma patients. Further research is needed to confirm whether this approach represents an improved technology for assessing cognitive processing in coma.

## 1. Introduction

In the context of brain-injured patients with disorders of consciousness (DoC), various auditory ERP components such as the MMN and P300 are considered useful predictors of emergence from coma [1,2,3] and key biomarkers in the information processing that may lead to conscious perception [4]. However, these responses are often absent or difficult to detect in coma patients at their classical scalp locations and time intervals as a result of white matter impairments and cortical dysfunction after severe brain injury [5]. Also, the presence or absence of these components is particularly challenging in intensive care settings due to the many sources of artifacts (e.g., the array of equipment at each ICU bed and/or the drastic reduction in the amplitude of brain signals), which make them difficult to isolate. Additionally, our prior work using longer testing periods than typically done clinically revealed that the MMN waxes and wanes in some comatose patients [6,7]. This discovery suggests that the literature showing the high positive predictive value of the MMN but its low sensitivity could be the result of this wax/wane phenomenon. It became apparent that this hitherto unrecognized “cycling” aspect of neurophysiological responses requires a method enabling the examination of sequential levels of neural processing from senses to responses, reflecting a conscious state such as the MMN.

Brain decoding approaches that incorporate machine learning tools in EEG/ERP data analysis have been increasingly embraced by cognitive neuroscience, and are potentially valuable in elucidating markers of neural processing (indexed by ERPs) and how they may differ between clinical and non-clinical populations. The recent advances of these methods have enabled researchers to conduct data-driven investigations wherein complex neural patterns in large datasets can be identified automatically, without relying on specialist expertise. Particularly, multivariate pattern analysis (MVPA), also referred to as multivariate decoding, is a technique mainly used to distinguish between experimental conditions based on the observed patterns of brain responses. It derives from the fields of pattern recognition and supervised machine learning, and is useful to track the temporal sequence of various levels of information processing, from sensory to high-level cognitive processes [8,9].

While traditional statistical methods such as t-test and analysis of variance (ANOVA) follow a univariate approach (i.e., taking information from single-recording channels separately or from averaged signals across multiple channels), MVPA takes into account the multivariate and high-dimensional nature of EEG by considering the distribution of activity from multiple channels simultaneously. This methodology provides a single-time curve of classification performance, which helps to overcome the inherent issues of statistical correction for multiple comparisons across channels, and therefore provides scientists with a rapid, interpretable signal at the whole-brain level with high temporal precision. Another interesting metric derived from MVPA can be used to characterize the temporal stability of the neural representations underlying different cognitive processes [10]. Several studies have reported that multivariate decoding analyses can predict survival rates in comatose patients [11,12,13], and automatically classify the patients’ level of consciousness [14].

In this study, we proposed the use of MVPA to discriminate the neural representations encoded in single-trial ERPs associated with auditory deviance detection in healthy controls, and showed evidence of its applicability in three comatose patients. We believe that this method is uniquely useful in characterizing longer than usual recordings to assess the waxing and waning of ERPs, as we observed in previous studies [6,7]. This prior work demonstrated the prominence of the waxing and waning cycles seen for the MMN. That is, within a longer recording there were clear periods of pronounced MMN (i.e., waxing) in coma patients and periods where, in the same patients, the MMN was absent or weak (i.e., waning). The objective here is to demonstrate as a proof of principle that coma-based “cycling” is not seen in healthy controls, and that there are sufficient “waning” periods in a coma that could account for the previous observations of low sensitivity in clinical studies [2,15]. Methodological aspects that influence classification performance, such as the signal amplitude and the number of electrodes, were also considered in order to evaluate the feasibility of this method for the assessment of brain activity underlying auditory deviance detection in comatose patients.

## 2. Materials and Methods

### 2.1. Data Collection, Preprocessing and Decoding Analysis

EEG/ERP data were collected from seventeen healthy controls (one 12 h recording each) and three comatose patients (two recordings of up to 24 h each). The healthy controls were 19 to 56 years old (mean = 29.64, standard deviation = 11.73) and had no history of neuropsychiatric disorders, alcohol/drug abuse, head trauma or known hearing impairment. For patients, two recordings were conducted 3 days apart, denoted as day 0 and day 3, respectively. Here, we analyzed the recording blocks of the auditory oddball paradigm designed to elicit mismatch negativity as part of a more extensive coma project [16]. The demographic and clinical information of patients are available in Table 1. Patients were off sedative medications during the EEG recordings. This included anesthetic agents such as propofol and small doses of benzodiazepines (e.g., midazolam) that were withheld for a minimum of 2 h prior to testing.

Continuous EEG data were recorded online (bandpass = 0.01–100 Hz and sampled at 512 Hz) using the Biosemi ActiveTwo system (Biosemi, Amsterdam, The Netherlands). For healthy controls, the electrodes were placed on the scalp according to the standard 10/20 system using a 64-electrode cap. A reduced number of 11 electrodes (F3, Fz, F4, C3, Cz, C4, P3, Pz, P4, T7, T8) following the same 10/20 system were used in the comatose patients due to surgical incisions and external ventricular drains (EVD). For all controls and patients, vertical and horizontal electro-oculogram (EOG) signals were monitored by electrodes placed above and over the outer canthus of the left eye, and reference electrodes were located bilaterally at the mastoids. Data pre-processing was conducted offline using Brain Vision Analyzer 2.1 (Brain Products Inc., Gilching, Germany). All recordings were re-referenced to the average mastoids and filtered (IIR Filters/Zero Phase Shift Butterworth) with a bandpass of 0.1–30 Hz (24 dB/oct; order 4) and a 60 Hz notch filter. Epochs containing non-ocular artifacts (e.g., muscle activity, movements) with values exceeding ±75 μV were removed. Eye movements and blinks artifacts were corrected using the Ocular Correction Independent Component Analysis (ICA) transformation. Infomax ICA was conducted on the scalp EEG channels (components = number of channels) and compared to EOG signals to select and reject the components most aligning with eye movements (typically n = 2 and corresponding to horizontal movements and blinks). Care was taken to avoid ICA computation with insufficient rank [17]. EEG trials were separated and segmented by stimulus type (i.e., duration deviants and standard tones) from 100 ms pre-stimulus to 600 ms post-stimulus, and baseline-corrected (−100 to 0 ms).

MVPA was then implemented to specifically quantify the differences in neural responses to standard vs. duration deviants in control subjects and comatose patients. The analyses that are presented here were performed using custom written-MATLAB scripts (MathWorks Inc., Natick, MA, USA) based on functions from the MVPA-Light toolbox [18] integrated in Fieldtrip [19].

To increase signal-to-noise ratio (SNR), all trials of up to five recording blocks of the oddball paradigm were concatenated to form a “superblock” per subject, which was then analyzed as a single subject dataset. Every healthy control had one superblock, while the comatose patients had two superblocks on day 0 and at least one superblock on day 3. For patients 2 and 3, the resulting superblock on day 3 contained two and six recording blocks respectively. This approach of concatenating a large number of trials was also carried out to reduce trial-to-trial variability within subjects, increase data size for training and provide a global characterization of individual differences between the auditory responses within a long period of time.

### 2.2. Classification Performance Across Time

Since oddball designs are often unbalanced, we first re-balanced the data by applying undersampling correction (i.e., removing trials belonging to the standard condition as the overrepresented class). The methods of under- or oversampling have been shown to prevent bias in classification, and improve (i.e., increase) the area under the curve (AUC), which is a widely-used quantitative measure of classification performance, calculated from the receiver operating characteristic (ROC) [20].

MVPA was then performed to discriminate between standard and deviant responses on 11 electrodes (F3, Fz, F4, C3, Cz, C4, P3, Pz, P4, T7, T8) common between patients and healthy controls as features for every time point separately, using a support-vector machine (SVM) classifier and the AUC as a performance metric. For SVMs, the C parameter was selected to maximize cross-validation performance in a search grid from 0.01 to 100 (see default in MVPA-Light toolbox). The input to the model was a 3-D (trials x electrodes x time points) matrix and a vector of class labels (i.e., standard and deviants).

MVPA preprocessing included z-scoring followed by a sample-averaging procedure as nested operations within a cross-validation analysis. A nested preprocessing essentially applies operations to the training and test sets separately. A nested z-scoring operation [18] implies that the means and standard deviations for the training data are computed, and therefore the training data are z-scored (normalized) accordingly. For the test data, the same mean and standard deviation were used to center and scale the data. As a result of the sample-averaging operation, trials from the same class were split into multiple groups, and subsequently the dataset was replaced by the group means to create new observations. The nested Z-scoring and sample-averaging operations were chained together (e.g., cfg.preprocess = ‘zscore’ ‘average_samples’) as described in [17], also available in Fieldtrip toolbox [19] and GitHub page [21]. Groups of 5 averaged trials were implemented for consistency across all subjects and patients, as increasing the number of trials improved slightly the classification performance, but led to a larger variability. A 5-fold cross-validation with 10 repetitions was implemented to get a more realistic estimate of the classification performance in discriminating the two classes of auditory responses. Lastly, a non-parametric permutation test was computed to assess whether the classifier performance (AUC) was significantly above-chance level (50%). An AUC of 50% is uninformative and implies random guessing, whereas a score of 100% amounts to perfect classification. The permutation test creates a null distribution by shuffling the class labels (i.e., standard and deviants) and repeating the multivariate decoding analysis multiple times. This permutation test was based on 100 random permutations, and corrected for multiple comparisons using the cluster statistic as described in [18] for Level 1 statistical analysis (i.e., single-subject analysis).

### 2.3. Generalization Across Time

An extension of the MVPA called “temporal generalization” was performed to explore whether the classifier’s performance can be generalized to time periods with shared representations [10,22]. That is, instead of training and testing the SVM classifier on data at each time point *t*, as described above, MVPA temporal generalization is tested across all possible time points *t*’. For example, if the classifier is trained to discriminate two activation patterns (e.g., standard versus duration deviant trials) at 200 ms and can successfully classify such patterns at 250 ms, one may infer that there is similar underlying neural representation (or cognitive processing) at those time periods. This analysis yields a 2-D temporal generalization matrix of cross-validated metrics. Conventionally, these matrices are depicted with the *y*-axis denoting training time, and the *x*-axis denoting testing time or generalization. In each cell of the matrix, the AUC scores are summarized. Classifiers trained and tested at the same time correspond to the diagonal of the matrix, which is referred to as “diagonal decoding” and closely corresponds to the outcome computed in the previous analysis (see Section 2.2). In contrast, if a matrix shows an off-diagonal pattern, it may indicate that the representation is stable (similar) over time or becomes reactivated at different times. Different generalization patterns can be distinguished across time (e.g., isolated, sustained, chained, reactivated patterns, and so on) [10].

Additionally, classification and temporal generalization analyses across time were run on each single recording block collected for each patient in order to identify fluctuations in decoding performance during the day. Since the number of trials is reduced in the single recording blocks compared to the superblocks, the sample averaging operation included a group of two averaged trials instead of five in the preprocessing.

### 2.4. Effects of Data Features on Decoding Performance

To evaluate whether the MVPA procedure is actually feasible for use in coma patients, we investigated the effects of the signal amplitude, as well as the selected number of the electrodes, on the classification performance in the control group.

Firstly, the mean amplitudes of each individual superblock were computed over 50 and 100 ms periods surrounding the peak latency of each component of interest, respectively (MMN, 80–230 ms and P3a, 250–350 ms), and correlated with the maximum AUC scores found within those latency intervals. Secondly, in order to assess whether the implemented decoding procedure (using 11 electrodes: F3, Fz, F4, C3, Cz, C4, P3, Pz, P4, T7, T8) preserved its predictive value for clinical purposes, we computed the same multivariate procedure using 64 electrodes in the control group, and compared the maximum AUC scores when reducing the number of electrodes from 64 to 11 using a paired-samples *t*-test. Finally, a “searchlight” approach with 5-k-fold cross-validation (as detailed in [18]), using a maximum of five ERPs (averaged responses) from each control subject, was carried out to identify which electrodes discriminated better between conditions across subjects. We hypothesized that the selected 11 electrodes contained sufficient discriminative information for the classification. This approach was implemented at the baseline (ranging from −100 to 0 ms) and in 50 ms intervals after stimulus onset (from 50 to 550 ms).

## 3. Results

### 3.1. Single-Trial Decoding in Control Subjects

All control subjects exhibited significantly above-chance performance after stimulus onset, peaking within the latency intervals associated with the MMN (150–230 ms) and the P3a components (250–350 ms). Maximum AUC scores when discriminating duration deviants versus standard tones ranged from 80% to 94%. A summary of the individual results is shown in Table 2. Notice that seven subjects exhibited maximum scores within the P3a interval.

**Figure 1:**
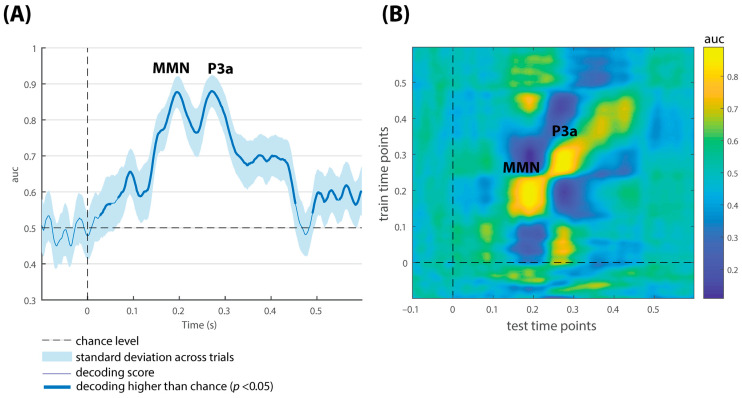
Multivariate decoding results of a representative control subject for duration-deviant vs. standard comparison. (**A**) Classification performance across time. The shaded area is the standard deviation across trials. The thick line indicates the time points where decoding is significantly higher than the chance level. (**B**) Temporal generalization plot of decoding performance. Color bar indicates AUC scores.

The multivariate decoding results from a representative control subject are displayed in Figure 1. Classification performance was significant after stimulus onset at the latency intervals associated with the MMN, the P3a, and to a lesser extent at time intervals preceding and following these components (see panel A). The diagonal of the temporal generalization matrix (in panel B) indicated a succession of processing stages, analogous to the classification performance curve (shown in panel A).

Given the variability across trials within each subject, off-diagonal transient generalization patterns (yellow patches) were observed, but two of them were consistently present in most of the subjects. As illustrated in the example (see Figure 1, panel B) the SVM classifier trained from 100 to 250 ms (corresponding to the time window of the MMN) generalized data at a later time, between 400 and 600 ms. Similarly, the training time points from 0 to 100 ms generalized around 250–300 ms.

### 3.2. Effects of Amplitude and Electrodes on Decoding Performance

**Figure 2:**
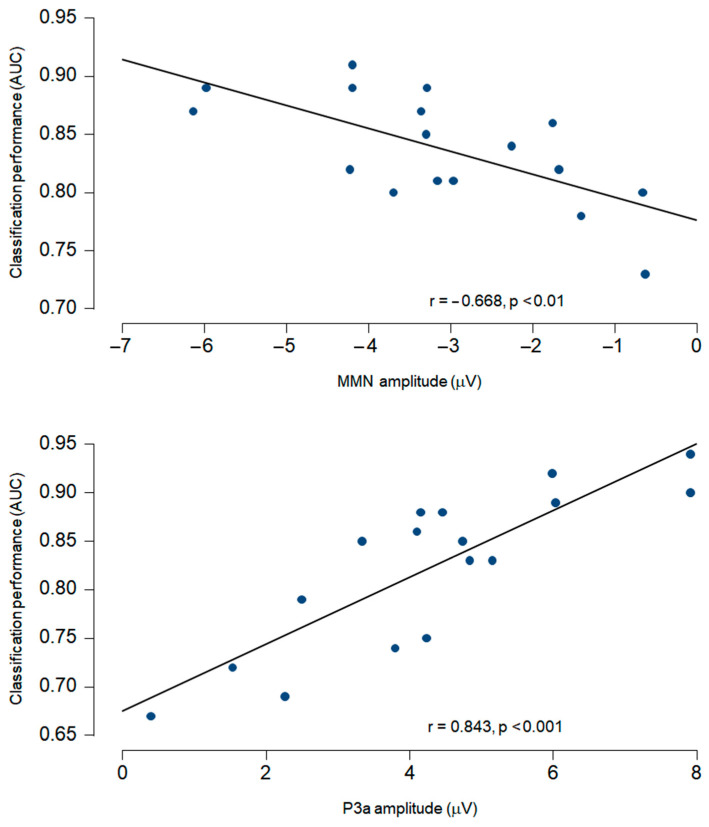
The correlation analysis between individual classification performance and ERP amplitude was significant for both the MMN and P3a components.

Using a Pearson correlation test, the classification performance was found to be strongly correlated with the amplitudes of both MMN (*r*(15) = −0.668, *p* < 0.01) and P3a components (*r*(15) = 0.843, *p* < 0.01) (see Figure 2).

**Figure 3:**
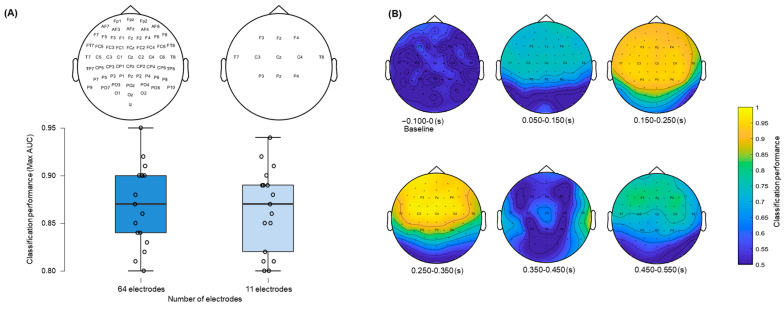
Effect of a reduced number of electrodes on classification performance and searchlight analysis across control subjects. (**A**) The paired-sample *t*-test revealed no significant differences in classification performance using 64 electrodes in comparison to 11 electrodes. (**B**) The searchlight MVPA computed over the baseline and 50 ms time intervals after stimulus onset showed the electrodes that better discriminated between conditions.

The effect of reducing the number of electrodes from 64 to 11 on the classification performance is shown in Figure 3. After confirming assumptions of normality (Shapiro–Wilk test = 0.908, *p* > 0.05), the paired-sample *t*-test showed no significant differences (*t*(16) = 1.49, *p* > 0.05), which suggests that the selected electrodes used in both controls and patients in the present study were sufficient to provide discriminative information between conditions. Figure 3 (panel B) showed the results of applying a searchlight MVPA approach across control subjects. Topographic maps showed high decoding performance during the time intervals associated with the MMN/P3a complex (150–250 ms; 250–350 ms), with maximum values at frontocentral electrodes. Notice that the selected 11 electrodes, which were the same as those used in coma patients, are spatially distributed on the regions showing the highest AUC scores.

### 3.3. Single-Trial Decoding in Coma

**Figure 4:**
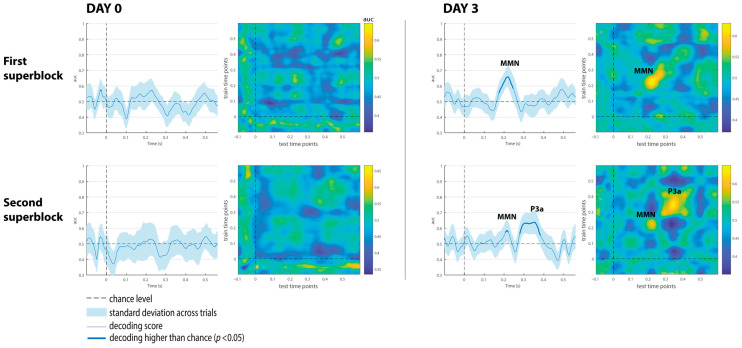
Multivariate decoding results of Patient 1 on day 0 and day 3. The shaded area (first and third columns) is the standard deviation across trials. The thick line indicates the time points where decoding is significantly higher than the chance level. Color bars in the temporal generalization matrices (second and fourth columns) indicate AUC scores.

**Figure 5:**
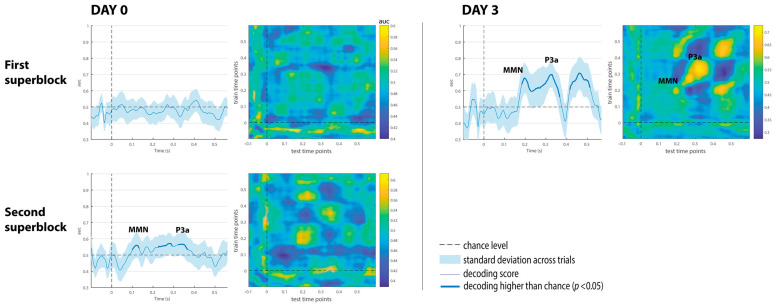
Multivariate decoding results of Patient 2 on day 0 and day 3. The shaded area (first and third columns) is the standard deviation across trials. The thick line indicates the time points where decoding is significantly higher than chance level. The color bar in the temporal generalization matrices (second and fourth columns) indicate AUC scores.

**Figure 6:**
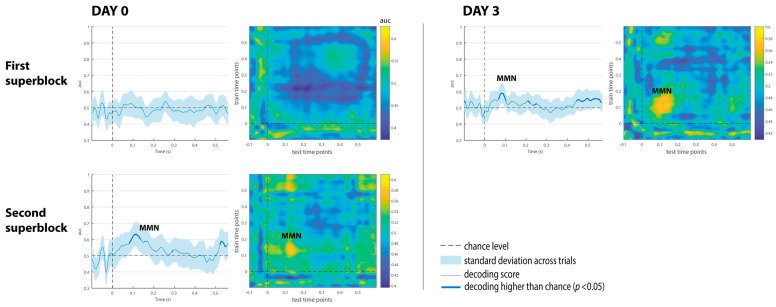
Multivariate decoding results of Patient 3 on day 0 and day 3. The shaded area (first and third columns) is the standard deviation across trials. The thick line indicates the time points where decoding is significantly higher than the chance level. The color bar in the temporal generalization matrices (second and fourth columns) indicates AUC scores.

The decoding results of the three comatose patients on each recording day are displayed in Figure 4, Figure 5 and Figure 6. While none of the comatose patients exhibited significant results at the first superblock on day 0, patients 2 and 3 displayed some intervals with significantly above-chance performance after stimulus onset at about the second half of day 0 (second superblock). Despite being reduced in comparison to controls, the classification performance was significantly above-chance on day 3 in all patients.

Patient 1 displayed significant decoding results in the two superblocks on day 3 (Figure 4). The first superblock showed maximum AUC scores around 65%, peaking at 205 ms, within the time window of the MMN component. The second superblock showed two consecutive intervals with above-chance performance (AUC scores around 59% and 62% respectively), suggesting the presence of the MMN/P3a complex. The temporal generalization matrices (on the right) consistently displayed these dynamical patterns of decoding performance on the diagonal.

Patient 2, who had shown reduced performance but a slightly higher than chance in the first recording session on day 0 (AUC scores ~56%), showed increased classification performance on day 3 up to 68, and 70% in intervals associated with the MMN and P3a components, respectively (see Figure 5). A later AUC peak at 71% was also observed around 460 ms. On that day, this patient showed behavioral signals of emergence from the coma state—spontaneous eye opening and withdrawal from pain, as reflected in a GCS score of 9. Based on the study protocol, only a superblock consisting of two recording sessions was performed in patients emerging from a coma. The temporal decoding matrix corresponding to day 3 showed several activation patterns on the diagonal, suggesting the presence of the MMN/P3a complex followed by a late pattern of discrimination between 400 and 600 ms. Transient generalization patterns (off-diagonal yellow patches) were also observed at time points above and below the diagonal, suggesting that brain generators might be reactivated at a later time.

Patient 3 displayed a maximum AUC of 63% around 120 ms at the second superblock on day 0, and an AUC of 60% at an early time interval, close to 100 ms on day 3 (see Figure 6). The time generalization decoding matrices revealed early decoding, limited to the diagonal associated with either the latency of the MMN or the DRN component (i.e., a deviant-related negativity, which represents a spatial–temporal summation of both N1 and MMN components), as described in [23].

### 3.4. Tracking Decoding Performance at Each Single Block in Coma

**Figure 7:**
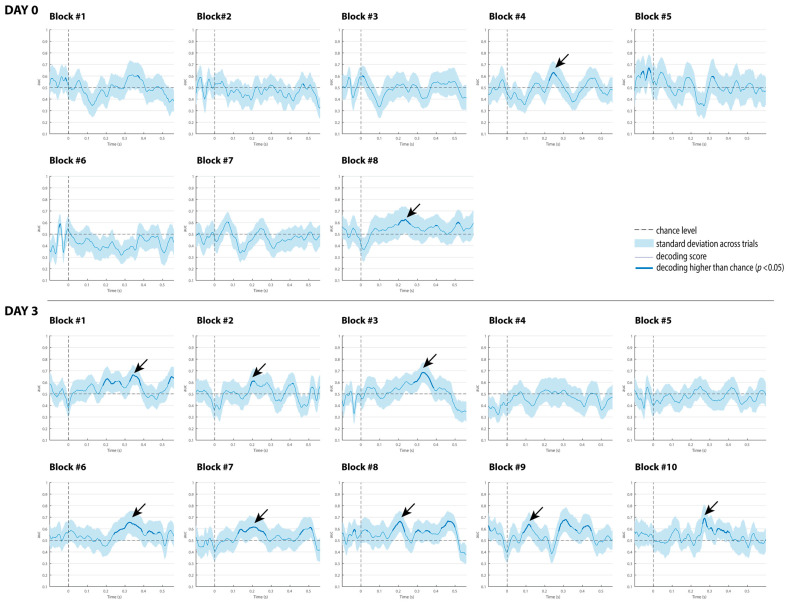
Classification performance of Patient 1 at each single block on day 0 and day 3. The shaded area is the standard deviation across trials. The thick line indicates the time points whereat decoding is significantly higher than chance level. Black arrows indicate the blocks with reliable classification performance.

**Figure 8:**
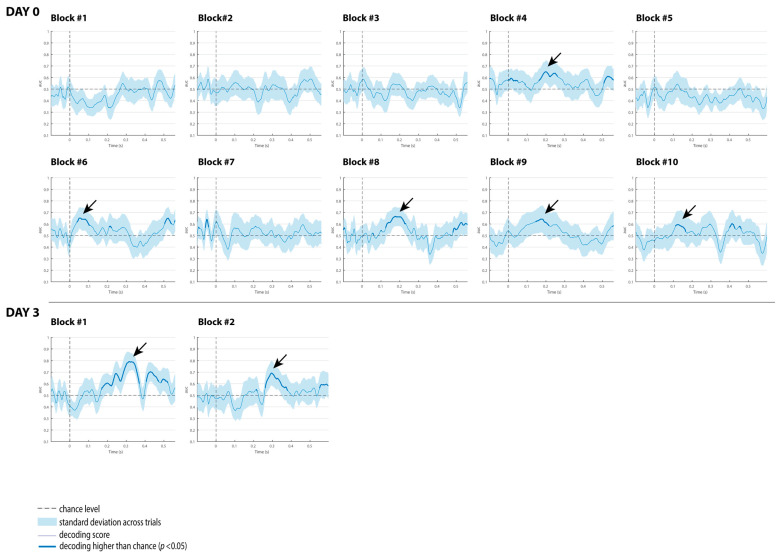
Classification performance of Patient 2 at each single block on day 0 and day 3. The shaded area is the standard deviation across trials. The thick line indicates the time points where decoding is significantly higher than chance level. Black arrows indicate the blocks with reliable classification performance.

**Figure 9:**
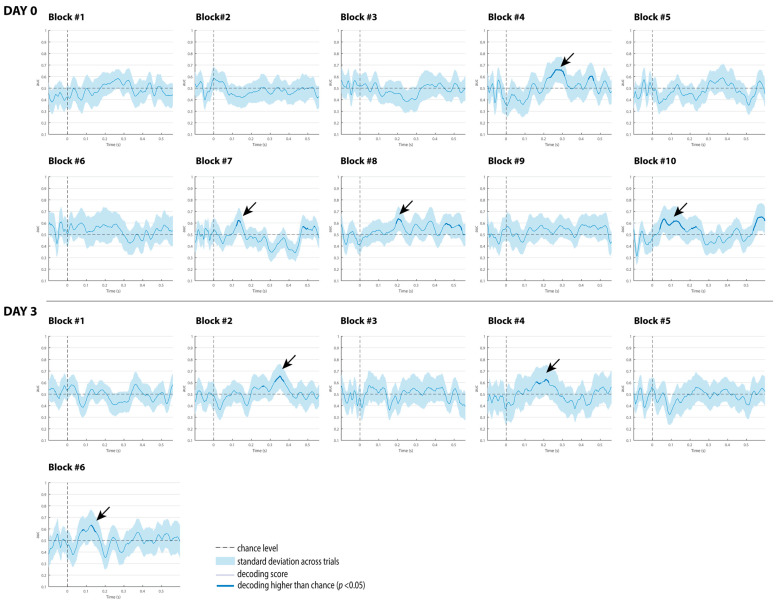
Classification performance of Patient 3 at each single block on day 0 and day 3. The shaded area is the standard deviation across trials. The thick line indicates the time points where decoding is significantly higher than chance level. Black arrows indicate the blocks with reliable classification performance.

The decoding results of the three comatose patients on each recording day are displayed in Figure 4, Figure 5 and Figure 6. For patients, the classification analysis across time was also computed at each single recording block to track decoding fluctuations over time, and to explore which single blocks contributed more to the global responses observed at each superblock. As displayed in Figure 7, Figure 8 and Figure 9, all patients showed significant decoding results (with AUC scores ranging from 62 to 65%) in only one block (block 4) out of the five single blocks that were used to form the first superblock on day 0.

Patient 1 had the highest number of single blocks with reliable classification performance on day 3, particularly in the last five blocks (blocks 6 to 10), which explains why the second superblock on day 3 showed higher AUC scores of 80 and 70%, respectively, in latency intervals associated with the P3a component (see Figure 8).

Patient 2 exhibited significant results in four single blocks at about the second half of day 0 (included in the second superblock). This patient showed signals of awakening from coma on day 3, and therefore only two single recording blocks were collected. Interestingly, these single recordings exhibited the highest scores of 80 and 70%, respectively, in latency intervals associated with the P3a component (see Figure 8).

In Patient 3, three blocks (7, 8 and 10) with significant results on day 0, and three blocks (2, 4 and 6) on day 3, were observed. Considering the proportions of blocks per patient, the classification performance for discriminating duration-deviants vs. standard sounds fluctuated (i.e., waxing and waning) across blocks in all patients, particularly in Patient 3 (Figure 9).

The three patients showed a slight behavioral improvement on day 3, as reflected by the clinical scales in Table 1. Despite the improvement in behavioral scales and decoding performance, Patient 1 passed away after withdrawal of life support. Patient 2, who awakened from coma on day 3, showed good recovery after a year. Patient 3 was discharged to a different hospital, remaining dependent on the ventilator, and subsequently transferred to a chronic care facility, where they were diagnosed as UWS.

## 4. Discussion

This study shows the dynamic waxing and waning of ERPs in a case series of comatose patients, and demonstrates that multivariate approaches are well suited to decoding single-trial ERP responses that have been typically associated with coma emergence. Our results show high decoding performance in all control subjects, with maximum AUC scores ranging from 80 to 94% corresponding to the MMN and P3a intervals. In some subjects (7/17), the AUC peaks were observed in the associated P3a intervals, a finding that has been previously reported and associated with an automatic attentional shift towards deviant sounds [24].

Here, we followed a similar approach to that used by Tzovara and collaborators, who applied a multivariate procedure in post-anoxic coma patients to decode the differences between deviant and standard trials elicited during a classic oddball paradigm [11]. In contrast to these authors, who modeled the distribution of single-trial ERP responses using a mixture of Gaussian assumptions, we used a linear support vector machine (SVM) classifier, and included nested operations, such as averaging trials within a cross-validation analysis. This averaging step seeks to increase the signal-to-noise ratio (SNR), which is known to improve the decoding performance [18,25]. These methodological differences, including the characteristic of the experimental paradigm and the fact that we looked at more single-time points, may explain why our approach achieved greater classification scores in healthy controls (mean AUC = 86%) in comparison to Tzovara et al.’s findings (mean AUC = 72%).

In a different context and using other active experimental tasks that required sufficient engagement and attention from the participants, King and colleagues found that single-trial classification with SVMs can achieve AUCs between 73% and 90% depending on the type of recording (EEG, intracranial EEG or magnetoelectroencephalography (MEG)), reaching mean AUC scores of 77.8% when recorded with high-density EEG [26]. Our findings confirm that the implemented decoding algorithm is robust and can automatically distinguish neural responses, but in passive listening conditions. While active paradigms are supported in the literature for their obvious benefits, they can also underrate the degree of consciousness in DOC patients. As reported in a meta-analysis, passive paradigms using fMRI or EEG suggested preserved consciousness more often than active paradigms in patients who emerged from coma as VS/UWS or MCS [27].

Additionally, the exploratory analysis of the temporal generalization matrices confirmed the presence of a serial chain of activation patterns associated with the presence of the MMN and the following P3a component that arises automatically shortly after the MMN. The authors of this method used MEG recordings while conducting an elegant variant of the classic oddball task, the local–global paradigm [28]. In this paradigm, two levels of regularity can be violated—one local, which implies a change of sound within each single trial, postulated to elicit the MMN/P3a complex, and one global (i.e., a change of sound sequence across trials) that generates the P3b component, which is thought to depend on working memory and conscious access. The activation patterns found in our sample, plotted in Figure 1 (panel B), are equivalent to the local effects found in previous research [10,29], in which the decoding of local standards versus local deviants led to the mismatch and the sequential recruitment of patterns of brain activity, as reflected in a diagonally shaped pattern of temporal generalization. Although there was considerable variability across trials within subjects, short off-diagonal patterns were also observed, two of them consistently seen in healthy controls. The classifier trained around 100–250 ms (the same time window of the MMN component) generalized data between 400 and 600 ms, and the training time points corresponding to 0–100 ms generalized over the latency related with the P3a component. Based on the main types of dynamics postulated by King and Dehaene, this phenomenon may indicate that similar generators associated with early processing stages could be reactivated at a later time, sharing common neural representations [10]. Future research, using source localization methods, could help to identify the brain generators of these ERP responses, and confirm whether they are reactivated.

### 4.1. Clinical Value for Coma Research

The decoding results of the three comatose patients on each recording day are displayed in Figure 4, Figure 5 and Figure 6. Decoding auditory ERP responses at the single-subject level promises to be of great value in clinical applications for coma patients. Consistent with previous evidence [24], the classification performance in our study was found to be strongly related to both the MMN and P3a amplitudes (Figure 3), suggesting that the analyses successfully capture relevant single-trial features in the data. In this regard, ERP-based decoding performance (i.e., AUC) could also be used as a neurophysiological tool for tracking amplitude fluctuations of brain activity. Another important aspect to take into account when implementing automatic decoding procedures is to determine the optimal number of electrodes to achieve high classification performance. Previous studies conducted in patients in acute coma state and other DoCs have implemented similar approaches using different EEG configurations, varying from 19 [11,12,30,31] to 128 electrodes [26]. While a high number of EEG electrodes raises computational and experimental costs, and might deteriorate the decoding performance because of the larger number of features involved, it has been found that reducing the number of EEG channels decreases the classification performance [24]. However, an important study conducted by Engemann and colleagues demonstrated that multivariate analysis, utilized to discriminate between levels of consciousness, is robust across different EEG configurations [32]. The authors found that discrimination performance increased with the number of electrodes and epochs, but was already strong with 16 electrodes, and with a 10–20 montage. In the current study, reducing all 64 available electrodes to 11 had no effect on the classification performance (Figure 3, panel A), which suggests that a few electrodes, located over midline, central and temporal regions, capture sufficient discriminative information of the auditory responses. Providing a robust decoding performance with such a reduced number of EEG channels is extremely useful for clinical applications in intensive care, since severe brain injuries and monitoring devices in some comatose patients may limit the placement of a high-density EEG montage on the scalp.

### 4.2. Decoding Single-Trials ERP Responses in Comatose Patients

Having demonstrated the feasibility of the decoding procedure in distinguishing between single-trial ERP responses in healthy controls, we applied it in three coma patients. The results reveal that while none of the patients exhibited significant results at the first superblock on day 0, two patients (Patients 2 and 3) showed some intervals with significant classification performance in about the second half on this day (i.e., second superblock). All of them exhibited maximum AUC scores of 65%, 70% and 60% in time windows associated with either the MMN or the P3a component on day 3. These findings are in line with previous work [11,12,31,33] that demonstrated intact auditory discrimination in the acute coma state, as well as important improvements in classification performance over time.

In contrast to these studies, none of the patients here underwent any therapeutic hypothermia treatment that could explain their improvement in auditory discrimination on day 3. We believe that the patients might have reflected positive neurophysiological changes after three days of intensive care, as illustrated in the slight increase in their behavioral scores (GCS and FOUR scores, in Table 1). This indicates that decoding performance may predict the patient’s clinical course. In fact, the patient with the highest decoding performance (Patient 2; AUC = 70%) showed behavioral signals related to coma awakening on day 3, and a positive outcome after a year of rehabilitation (i.e., good recovery). Similar to controls, the temporal dynamical activation patterns in this patient were observed in a chain (although slightly isolated) on the diagonal of the temporal matrix, with transient generalization patterns (off-diagonal). Additionally, the multivariate analysis applied to the single-recording blocks showed a maximum AUC score of 80% on day 3 (Figure 8). Such an increase in decoding performance in a brain-injured patient in comparison to other single blocks provides further evidence of the waxing–waning pattern underlying the conscious state (not necessarily awareness) in a coma [6]. It also highlights the potential use of this decoding technique to track the progression of auditory discrimination and complement other available tests and behavioral assessments to predict coma emergence. As stated by Morlet and Fischer in a comprehensive review, multivariate algorithms can provide the assessment of sensory and mismatch processes without classical ERP component identification or a priori hypotheses about such components [2].

The other two patients showed different outcomes; one was considered in need of palliative care and passed away after the withdrawal of life support (Patient 1), while the other (Patient 3) survived in the ICU, but was eventually declared to be in VS/UWS and transferred to a chronic care facility. These results are also coherent with Tzovara’s findings that showed evidence that both coma survivors and non-survivors exhibited accurate auditory discrimination during the acute coma state, and only their positive or negative progression in sound discrimination over time was the major predictor of their chances of survival and outcomes [11,12]. Based on this scenario, we cannot discard the possibility that Patient 1 would have survived and met a different outcome if the critical care had not been withdrawn, especially after showing consistent improvement in auditory discrimination on day 3. In Canada, the withdrawal of life supporting interventions is a common cause of death in 70% of patients with DOC, and end-of-life decisions often preempt accurate prognostication [34]. Those family and medical decisions are based, to some extent, on the fact that many survivors of severe brain injuries who emerge from coma may remain in either VS/UWS or MCS, with physical and cognitive disabilities, for decades, or the rest of their lives [35].

Importantly, the “waning” patterns, particularly observed within the MMN intervals in patients who do not emerge from coma, may account for the previous reports of low sensitivity in clinical studies [2,15] that tested MMN on one occasion only. That is, those studies could have investigated a patient during a “waning” of MMN, and thus provided a misleading assessment of that patient and their likelihood of emerging from the coma. Moreover, there are several etiologies that lead to coma, and establishing if this cycling of MMN varies across different types of coma would be valuable in terms of prognostics. The rate of cycling (e.g., a single wax/wane cycle every 8–10 h versus 2–4 h) might also have implications for predicting when emergence will occur, and what the patient’s diagnosis may be upon emergence (e.g., slow cycling may be linked to unresponsive wakefulness syndrome (UWS) while rapid cycling may be linked to a locked-in state). However, much larger samples will be required to evaluate these hypotheses.

Our findings can also be contextualized with respect to a recent report showing that single-trial neural responses (i.e., phase locking) to auditory stimuli can be predictive of coma outcome [36]. Notably, this study also utilized an auditory oddball paradigm, but did not directly focus on ERP features, and provided results suggesting that the processing of auditory stimuli may distinguish between surviving and non-surviving coma patients. Our present results suggest that more frequent testing (as opposed to the 21 min protocol in [36]) may augment the study’s sensitivity in terms of positive outcome prediction.

Although our small sample limits the generalization of our findings across the patient population, we observed robust responses on a single-trial basis within each subject. These preliminary results, using a single-subject design, encourage the repetitive use of neurophysiological measures along with decoding methods to support the provision of life-sustaining therapies, particularly when there is clinical uncertainty of coma emergence.

## 5. Conclusions

Our multivariate decoding approach revealed that the neurophysiological responses elicited by duration-deviants and standard sounds during an oddball paradigm can be robustly discriminated in healthy controls. The same approach applied to a small sample of comatose patients showed lower decoding performance in comparison to controls, but the response was still significant, mainly during the second recording day. If assessed in larger patient cohorts, our findings may have potentially important clinical implications, because they could provide clinicians with an automatic tool to monitor neurophysiological changes over time, and improve the prediction of coma outcome.

## Figures and Tables

**Table 1:** Demographic and clinical information of patients.

Patient	Sex	Age	Etiology	Testing Day	State	Days Since Coma Onset	GCS ^1^ (E,V,M)	FOUR ^2^ (E,M,B,R)	Blocks Recorded	Outcome
1	F	41	Neurosurgery	0	Coma	20	5 (1,1,3)	6 (0,1,4,1)	8	Death
				3	Coma/UWS	23	6 (4,1,1)	8 (3,0,4,1)	10	
2	F	51	Neurosurgery	0	Coma	8	5 (1,1,3)	5 (0,1,4,0)	10	Good recovery
				3	Awakening	11	9 (4,1,4)	9 (3,1,4,1)	2	
3	F	43	Trauma	0	Coma	13	4 (1,1,2)	5 (0,1,4,0)	10	UWS ^3^
				3	Coma	16	7 (2,1,4)	5 (0,1,4,0)	6	

^1^ GCS: Glasgow Coma Scale, which is the sum of E (eye opening), V (verbal response) and M (motor response) scores. ^2^ FOUR: Full Outline of Unresponsive score that has four components—E (eye response), M (motor response), B (brainstem reflexes) and R (respiration). ^3^ UWS: Unresponsive wakefulness syndrome.

**Table 2:** Summary of maximum AUC scores for each individual control subject, including standard deviation (SD), latency, and the associated ERP components.

Subjects	AUC (%)	SD (%)	Latency (s)	ERP
1	0.94	0.03	0.294	P3a
2	0.82	0.04	0.156	MMN
3	0.81	0.06	0.154	MMN
4	0.86	0.05	0.162	MMN
5	0.90	0.03	0.269	P3a
6	0.89	0.05	0.255	P3a
7	0.88	0.04	0.271	P3a
8	0.80	0.06	0.212	MMN
9	0.85	0.85	0.267	P3a
10	0.87	0.05	0.222	MMN
11	0.89	0.03	0.195	MMN
12	0.91	0.05	0.212	MMN
13	0.89	0.06	0.208	MMN
14	0.80	0.07	0.199	MMN
15	0.92	0.06	0.267	P3a
16	0.85	0.06	0.271	P3a
17	0.81	0.11	0.201	MMN

## Data Availability

The original contributions presented in the study are included in the article. Further inquiries can be directed to the corresponding author.
